# *Dermanyssus gallinae *in layer farms in Kosovo: a high risk for *salmonella *prevalence

**DOI:** 10.1186/1756-3305-4-136

**Published:** 2011-07-15

**Authors:** Afrim Hamidi, Kurtesh Sherifi, Skender Muji, Behlul Behluli, Fatgzim Latifi, Avni Robaj, Rezart Postoli, Claudia Hess, Michael Hess, Olivier Sparagano

**Affiliations:** 1 Faculty of Agriculture and Veterinary, University of Prishtina, Kosovo; 2 Faculty of Veterinary Medicine, Tirana, Albania; 3 Clinic for Poultry, Fish and Reptiles, Veterinary Medical University Vienna, Austria; 4 Northumbria University, School of Life Sciences, Newcastle upon Tyne, UK

## Abstract

**Background:**

The poultry red mite (PRM), *Dermanyssus gallinae (D.g.) *is a serious ectoparasitic pest of poultry and potential pathogen vector. The prevalence of *D. g*. and the prevalence of *Salmonella *spp. within mites on infested laying poultry farms were investigated in Kosovo.

**Findings:**

In total, 14 populated layer farms located in the Southern Kosovo were assessed for *D. g*. presence. Another two farms in this region were investigated 6 months after depopulation. Investigated flocks were all maintained in cages, a common housing system in Kosovo. A total of eight farms were found to be infested with *D*. *g*. (50%) at varying levels, including the two depopulated farms. The detection of *Salmonella *spp. from *D. g*. was carried out using PCR. Out of the eight layer farms infested with *D. g*., *Salmonella *spp. was present in mites on three farms (37.5%).

**Conclusions:**

This study confirms the high prevalence of *D. g*. in layer flocks in Kosovo and demonstrates the link between this mite and the presence of *Salmonella *spp. on infested farms.

## Background

The PRM *D*. *g*. is one of the most serious and widespread pests of poultry production. It is a worldwide ectoparasitic pest [1], showing genetic variation between the UK, France and Italy [2]. *D. g*. hides in cracks and crevices in poultry units and infests the birds only briefly for blood meals, mainly at night [3]. This haematophagous mite feeds rapidly from its host (in comparison with ticks for example) and can survive several months without a blood-meal. The mites enter poultry houses through open wall fans, wall inlets and air chimneys [4] or are brought by staff, bird cages, crates or wild birds. In recent years, the frequency of *D. g*. infestations in laying hens has increased in Europe, as has its pest status.

*D. g*. can have a significant economic impact in poultry production by causing a reduction in egg production, loss in body weight of birds, and a reduction of welfare of laying hens [5]. *D. g*. may also cause dermatitis in humans [6,7]. Infestation with *D*. *g*. can cause irritation and restlessness in affected hens and can even result in the death of birds [8]. It is reported that the death rate among the hens can rise from 1 to 4%, with a reduction in laying performance of up to 10%, as a result of infestation. Downgrading of egg quality in poultry affected by *D*. *g*. has also been observed [9,10].

In addition to causing 'direct' losses in poultry production systems, *D. g*. has also been described as a potential vector of several bacteria and viruses of concern to poultry, among them several food borne pathogens [11-14].

In order to assess levels of *D. g*. infestation on farms different mite traps have been evaluated and used as indicators to inform control decisions [15,16]. Effective options available for control of *D. g*. are limited [17]. The use of inadequate, ineffective, or even illegal chemicals have been responsible for increases in infestation rates due to the spread of acaricide resistance now common in many countries [1]. Nevertheless, some products are still being developed and used with some success. For example, [18] found that phoxim 50% is an effective acaricide against *D*. *g*. with [19] showing the same to be true of spinosad.

*D. g*. is widespread among farms in Kosovo and might cause high economic losses within the national poultry industry.

## Infestation rate

Fourteen populated layer farms (6,000 - 20,000 birds, from 6 to 12 months of age) and two de-populated farms located in Southern Kosovo were investigated for the prevalence of *D. g*. (Table 1). The 16 farms all had generally poor biosecurity measures, such as lack of traffic control and personal hygiene.

**Table 1:** Data on *D. g*. samples from poultry farms in Kosovo

Farm	1	2	3	4	5	6	7	8
nr. of samples	10	10	10	10	10	10	10	10
nr. of PRM/sample	50	50-100	100	50	50	50	50-100	50-100
layers on the farm	No	Yes	Yes	Yes	Yes	Yes	Yes	No
nr. of layers on the farm	0*	6,000	12,000	15,000	20,000	18,000	16,000	0
Salmonella PCR	+	+	-	-	-	-	-	+

*Depopulated more than 6 months before sampling

*D. g*. prevalence was determined using cardboard traps, which were placed into cages, but out of reach of the birds. Traps were left in place for 48 h, after which they were removed and evaluated for the presence of *D.g*. Ten cardboard traps were used at each farm and combined as a single pooled sample on the day of their collection. Traps, which were positive for *D. g*., were frozen at - 20°C to kill any mites present. Mite numbers were assessed and the number of individuals (adults, larvae and nymphs) per trap were estimated to the nearest 50.

## Incidence of *Salmonella *spp

Pooled samples of *D. g*. adults, nymphs and larvae (of between 50 and 100 mites) from each infested farm (n = 8), were selected in the laboratory and placed into plastic tubes and stored at -20°C. All samples (n = 80) of *D*. *g*. were examined for the presence of *Salmonella *using PCR.

Bacterial DNA was extracted using Dneasy Tissue Kit (QIAGEN) according to the manufacturer's instructions. Extracted DNA (25 μl/pooled mite sample) was stored at - 20°C. The PCR reaction mixture (25 μl) contained: 5 μl DNA sample, 14.05 μl distilled H_2_O, 2.5 μl 10xPCR Buffer, 0.75 μl MgCl_2 _(50 mM), 0.5 μl dNTP (10 mM), 1 μM of each primer and 1.25 U Taq DNA Polymerase. DNA was amplified according to [20]. The 16S rDNA primer sequences were: (forward)5'-TGT TGT GGT TAA TAA CCG CA-3' and (reverse)5'-CAC AAA TCC ATC TCT GGA-3'. PCR amplifications were performed in thermal cycler with a cycling program consisting of a 10 min denaturation step 94 C, followed by 35 cycles of denaturation (1 min, 94°C), annealing (45 s, 55°C) and extention (1 min 30 s, 72°C) and final extention step of 10 min at 72°C [21]. After 35 cycles the amplification product was expected to be 574 base pairs. Amplification products, including their size, were determined by electrophoresis using 2% agarose gels.

## Results and discussion

In total 8 out of 16 farms (50%) were infested with *D. g*. to varying degrees, where mites were present in both populated and unpopulated units. Out of the eight infested farms, pooled mite samples from three premises were positive for the presence of *Salmonella *spp. (37.5%). In two cases *Salmonella *was found in mites 6 months after the removal of layers (Farms 1 and 8). The other *Salmonella*-positive case was from mites trapped in a populated unit (Farm 2). Results are shown in Table 1.

*D. g*. represents a major problem for the layer industry in Kosovo and across much of the globe. Data from the present study showed that 50% of Kosovan cage rearing systems were infested with *D. g*., which is comparable to prevalence figures in other countries with free range rearing systems, e.g. France (56%), UK (60%), and Denmark (68%) [1]. A high *Salmonella *spp. prevalence on infested farms was also observed, which demonstrates that *D*. *g*. may at least carry this pathogen potentially serving as vector for its spread in poultry. Such a vectorial role has already been observed under laboratory conditions [22].

As mites tested positive for *Salmonella *spp. in depopulated units, the role that *D. g*. can play in between-flock transmission of pathogens was also demonstrated by the present study. *Salmonella *was found within *D. g*., 6 months after the removal of birds from the infested farm. This further suggests that transovarial transmission of *Salmonella *by *D. g*. is a possibility considering that mites do not survive for so long they must have passed the infection to their offspring. These same results show that *D. g*. can survive long periods of fasting, suggesting that these mites may be capable of pathogen transmission between flocks. Finally, where *Salmonella *was found in mites present on a populated farm, no signs of clinical Salmonellosis were observed in the birds. This could pose a risk to public health through Salmonella-positive mites being squashed on eggs or when such mites would infect birds.

The transfer of *D. g*. in Kosovo seems to occur via egg boxes which are exchanged at supermarkets, originating from different egg supply companies. In general all farms infested with *D. g*. in the present study demonstrated poor biosecurity measures. The emerging potential role of *D. g*. as a vector for *Salmonella *spp. supported by the results presented in this paper it should encourage the poultry industry to initiate strict biosecurity programs to control both D. g. and any pathogens it may foster, carry or transmit. Assuming that these results are not specific to Kosovo, they could explain why salmonellosis outbreaks are still observed in other countries, such as the UK. Although all birds are vaccinated against *Salmonella *the prevalence of *D. g*. is very high in the UK, which could explain why *Salmonella *transmission can overcome the vaccination effect as different serovars exist.

## List of Abbreviations

D.g.: *Dermanyssus gallinae*; PRM: poultry red mite.

## Competing interests

The authors declare that they have no competing interests.

## Authors' contributions

AH worked on study design, sampling, data analysis and drafting the paper. KS participated in sampling, carried analysis together with CH at the clinic of Poultry, Reptiles and Fishes at the Veterinary Medical University in Vienna. MH worked on study design, data analysis and drafting the paper. SM, BB, FL, AR and RP participated in sampling of *D. g*. OS worked on the data analysis and drafted the paper with AH. All authors read and approved the final version of the manuscript.
